# Integrated SegFlow, µSIA, and UPLC for Online Sialic Acid Quantitation of Glycoproteins Directly from Bioreactors

**DOI:** 10.1002/elsc.202400031

**Published:** 2025-01-23

**Authors:** Letha Chemmalil, Tanmay Kulkarni, Mathura Raman, Priya Singh, Yueming Qian, Chris Chumsae, Kyle McHugh, Zhuangrong Huang, Eric Hodgman, Michael C. Borys, Julia Ding, Gloria Li, Anthony Leone

**Affiliations:** ^1^ Biological Process Analytical Group Bristol Myers Squibb Devens Massachusetts USA; ^2^ Analytical Science & Technology Bristol Myers Squibb Devens Massachusetts USA; ^3^ Horizon Therapeutics Bristol Myers Squibb Rockville Maryland USA; ^4^ Analytical Development & Analytical Attribute Science in Biologics Bristol Myers Squibb Devens Massachusetts USA; ^5^ Takeda Pharmaceuticals Former BMS Affiliate Lexington Massachusetts USA; ^6^ Process Development Group Bristol Myers Squibb Devens Massachusetts USA; ^7^ Vertex Pharmaceutical Bristol Myers Squibb Boston Massachusetts USA

**Keywords:** µSIA, continuous bioprocessing, FIA labs, monoclonal antibody (mAb), process analytical technology (PAT), quality by design (QbD), SegFlow

## Abstract

This study emphasizes the critical importance of closely monitoring and controlling the sialic acid content in therapeutic glycoproteins, including EPO, interferon‐γ, Orencia, Enbrel, and others, as the level of sialylation directly impacts their pharmacokinetics (PK), immunogenicity, potency, and overall clinical performance due to its influence on protein clearance via hepatic asialoglycoprotein receptors (ASGPR). The ASGPR recognizes and binds to glycoproteins exposed to terminal galactose or N‐acetylgalactosamine residues, leading to receptor‐mediated endocytosis. Recent studies have demonstrated that sialylation of O‐linked glycan plays a role in protecting against macrophage galactose lectin (MGL)‐mediated clearance. In addition to the impact on serum half‐life, sialylation can influence other clinical outcomes, including immunogenicity, potency, and cytotoxicity. Therefore, the level of sialic acid is a critical quality attribute (CQA), and monitoring and regulating sialylation has become a regulatory requirement to ensure desired clinical performance. To achieve consistent levels of sialic acid‐to‐protein ratio, the time of upstream harvest and conductivity of downstream wash buffers must be tightly regulated based on the sialic acid content. Therefore, the utilization of process analytical technology (PAT) tools for generating real‐time or near‐real‐time sialic acid content is a business‐critical requirement. This work demonstrates the utility of an integrated PAT system for near real‐time online sialic acid measurements. The system consists of a micro‐sequential injection analyzer (µSIA) interfaced with SegFlow and an ultra performance liquid chromatography (UPLC). The fully automated architecture exemplifies the execution of online sampling, automatic sample preparation, and subsequent online UPLC analysis. This carefully orchestrated PAT framework effectively supports the requirements of QbD‐driven continuous bioprocessing.

## 1 Introduction

Glycoproteins comprise the majority of biotherapeutic drugs, contributing to the billions of dollars in worldwide sales of biotherapeutics [1]. These glycoproteins, produced in living cells using recombinant DNA (rDNA) technology, must be regulated for post‐translational glycosylation. Sialylation is critical for maintaining desired pharmacokinetics and pharmacodynamics (PK/PD) to achieve improved serum half‐life, bioactivity, and protein stability. The PK of protein therapeutics is influenced by sialylation to prevent the galactose moiety from binding to the hepatocyte asialoglycoprotein receptor (ASGPR) and reducing glomerular clearance [2]. Terminal sialic acid blocks the binding of glycoproteins to the hepatocyte ASGPR and prevents the clearance of IgG from circulation [3]. When the sialic acid is removed, the exposed galactose residue binds to the ASGPR found on mammalian hepatocytes. Asialoglycoprotein with exposed galactose is removed from circulation through receptor‐mediated endocytosis (Steirer 2009).

**Summary**

- Level of sialic acid is a CQA for fusion protein‐therapeutics due to its impact on PK/PD. Therefore, a mole‐to‐mole ratio of protein to sialic acid must be maintained within specification.
- Current industrywide practice is to execute harvest when the sialic acid level is closer to the specification range. The lengthy turnaround time of offline sialic acid testing makes it difficult to orchestrate precise harvest decision based on test results.
- Online testing utilizing integrated µSIA interfaced with SegFlow and UPLC can enable the generation of near‐real‐time results to make a timely harvest decision.
- We have demonstrated that the online results generated using this automated workflow are comparable to the offline results.
- With the elimination of human intervention, the online sialic acid method provides a better precision than the offline method. We believe that sharing this integrated practical solution may help others in the biopharma sector to overcome this industry‐wide challenge.

It has been demonstrated that the sialylation of terminal galactose residues of glycoproteins such as EPO, interferon α, interferon γ, and IgG prevents endocytosis and provides a prolonged circulatory lifetime [2]. Examples of other glycoproteins in which sialic acid plays an essential role include α‐2‐macroglobulin, ceruloplasmin, follitropin, α‐1‐acid glycoprotein‐1, hemopexin, lutropin, lactoferrin, anti‐HER‐2, interferon alpha‐2b, hTSH, tFSH, and Fc‐CTLA‐4 (Chia 2023). During the manufacturing process of recombinant glycoproteins, loss of sialylation occurs due to sialidase activity and should be closely monitored and controlled [4]. The reduced half‐life of protein therapeutics in the absence of sialic acid is governed by the clearance of galactose‐exposed asialoglycoproteins via ASGPR‐mediated endocytosis (Chemmalil 2014). Sialylations of the Fc domains of IgG also help to improve solubility, anti‐inflammatory activity, and thermal stability [3]. O‐linked sialylation may also protect against rapid clearance of glycoproteins from circulation by inhibiting macrophage galactose lectin (Ward 2022).

Sialic acids are negatively charged terminal monosaccharides attached to galactose residues at the nonreducing termini of both N‐ and O‐linked glycans. The two most common sialic acids in biotherapeutics are N‐acetylneuraminic acid (NANA) and N‐glycolylneuraminic acid (NGNA). While NANA is found in both human and non‐human cells, NGNA is synthesized by all mammalian cells except for human cells [5]. The presence of terminal NANA has been shown to impact various properties of glycoproteins, including circulatory half‐life, solubility, and thermal stability [6]. Engineered EPO with hyper‐sialylation has been shown to exhibit better pharmacokinetic properties (Varki and Schauer 2009). The difference of one oxygen atom between NANA and NGNA is sufficient for NGNA to be highly immunogenic in humans [7]. Hence, maintaining the desired levels of NANA and NGNA in protein therapeutics is crucial to ensure desired safety and PK/PD. The presence of the sialic acid derivative Neu5,9Ac2 on the protein surface tends to improve circulatory half‐life [8].

To maintain a desired level of sialic acid, it is crucial to carefully design and control the amount of sialic acid attached to the protein molecule (Varki and Schauer 2009). If the sialic acid content of a therapeutic protein in harvest cell culture fails to meet the established specification, the batch must be discarded unless the sialic acid enrichment is performed during the downstream unit operations (Dahotre 2022). To execute the harvest decision based on sialic acid content, generation of real‐time/near real‐time sialic acid data is essential for upstream cell culture. Similar process control is required during the downstream purification steps such that the conductivity of the wash buffer can be adjusted in accordance with the sialic acid content of the loading material. To decide on the ionic strength of the wash buffer, an accurate assessment of sialic acid content at the termination of the preceding step of the unit operation is a prerequisite. To maintain the sialic acid‐to‐protein ratio, rapid generation of sialic acid results, as well as the synchronization between the PAT and the process equipment, is critical [6].

Allison et al. [9] argued that the deployment of PAT tools not only improves process understanding but also provides the opportunity to control the process to ensure consistent product quality. Regulatory agencies are in favor of QbD‐enabled continuous bioprocessing to secure desired product quality and batch‐to‐batch reproducibility [10]. These agencies encourage drug manufacturers to embrace QbD and PAT technologies for safe and effective drug manufacturing [11, 12] (Alper and Rapporteur 2019). The FDA encourages drug manufacturers to deploy PAT tools for the timely measurement and control of CQAs to ensure desired final product quality [13]. Health regulators are urging the use of advanced PAT tools to establish design spaces to enhance process understanding and timely process control to manufacture high‐quality biologic drugs [14].

Shifting from batch processes to QbD‐enabled continuous bioprocessing with the deployment of PAT tools not only provides an opportunity to enhance process and product understanding but also facilitates cost‐effective manufacturing [15, 16]. QbD‐driven continuous bioprocessing offers efficient delivery of high‐quality products at a reduced cost [17]. The shift from the conventional quality‐by‐testing paradigm to the emerging quality‐by‐design approach requires the deployment of PAT tools. As Rathore and Winkle [12] stated, the implementation of PAT is critical for developing safe and efficacious therapeutics. Modernized drug manufacturing allows companies to leverage integrated PAT tools instead of testing product quality at the end of the process [18]. This is in alignment with the FDA‐recommended QbD‐based drug development and manufacturing to improve product quality [19].

Biopharmaceutical companies are transitioning to adopt the QbD paradigm instead of a quality‐by‐testing strategy to increase product and process understanding to comply with the FDA's guidance [20]. Implementing a QbD strategy instead of traditional quality‐by‐testing practices is intended to improve product quality and productivity [14]. Drug manufacturers are encouraged by health authorities' guidance to take advantage of the QbD and PAT platform for new process approvals [11]. Continuous bioprocessing can dramatically shorten the time it takes to scale up manufacturing for newly approved drugs (Alper and Rapporteur 2019). Process monitoring using online analytical techniques will lead to efficient process development and control (Alper and Rapporteur 2019). Timely monitoring and controlling of process parameters that influence glycosylation enable the achievement of a desired glycosylation [21].

Despite the numerous advances that have been made, the field of PAT is still undergoing evolution in the context of methodological and technological innovations [22]. To support the FDA's vision on the topic of efficient and flexible continuous manufacturing, biopharmaceutical companies are eager to adapt the emerging PAT platform [23]. PAT tool development for biopharmaceutical applications is more complex than developing PAT tools for conventional pharmaceutical operations, due to challenges posed by the large size of proteins and the dynamic nature of complex matrix components of the in‐process samples. By using an appropriate sampling device in conjunction with 1D and 2D‐LC (one‐dimensional and two‐dimensional liquid chromatography), these challenges can be overcome to achieve online measurements of CQAs and CPPs of upstream and downstream in‐process samples [24, 25, 26, 27].

Since sialic acid has a significant effect on PK, it is critical to regulate sialylation during the production of glycoproteins [28]. To this end, biotherapeutic manufacturers have made enormous efforts to ensure that the approved drugs fall within the defined sialic acid specifications [1]. Various methods have been established for sialic acid quantitation, including HPLC, plate‐based colorimetric/fluorometric assays, and LC/MS‐based methods. For HPLC analysis, the free sialic acid is often derivatized with 1,2‐diamino‐4,5‐methylenedioxybenzene (DMB) for fluorescence‐based detection. For the quantitation of non‐derivatized sialic acid, LC/MS is a viable option but may not be suitable for routine release testing. HPLC interfaced with an NQAD detector has been demonstrated to be a suitable alternative [29]. Over the past decade, several bioaffinity‐based approaches for the direct detection of sialic acids have been developed [30].

The effective application of QbD and PAT in real‐time monitoring and controlling of glycosylation truly enables successful drug development [31]. The motivation behind the study presented in this paper is to explore the use of a sequential µ‐injection analyzer (µSIA) system, controlled by a Python algorithm, for the pursuit of online sialic acid quantitation. The current practice of offline sialic acid quantitation at the end of the cell culture process exhibits a bias due to the lag time associated with the lengthy analysis time. A similar bias occurs during the downstream purification step, where the molarity of the wash buffer is calculated based on the sialic acid content of the feed material. The use of an integrated µSIA system as a sample preparation platform, coupled with the SegFlow sampling device and an integrated online ultra performance liquid chromatography (UPLC) system, facilitates online monitoring and controlling of sialic acid to maintain a consistent sialic acid‐to‐protein ratio from batch to batch.

## 2 Materials and Methods

### 2.1 Reagents

#### 2.1.1 Reagents, Columns, and Systems

InfinityLab Poroshell 120 EC‐C18 column (2.1 × 75 mm, 2.7 µm) was purchased from Agilent. Ascentis Express 90A, RP Amide columns (10 cm × 2.1 mm, 2.7 µm; 10 cm × 2.1 mm, 2 µm) were purchased from Sigma‐Aldrich. The sialic acid kit, containing DMB, phosphoric acid, NANA, NGNA, and fetuin, was purchased from Agilent. HPLC‐grade water and acetonitrile for the mobile phases were purchased from Fisher Scientific (Hampton, NH), and formic acid was purchased from Sigma‐Aldrich (St. Louis, MO). All chromatographic separations were performed on a Waters ACQUITY Classic UPLC system composed of the following modules and control software: Binary Solvent Manager, Sample Manager, Column Manager (CM‐A), UV/Vis/fluorescence detector, and Empower‐3 Software/Windows 10 PC. Note: The µSIA cannot work with sample managers with flow‐through needles (FTN) because the injection valve on an FTN injector lacks the necessary ports to allow a sample to be fed from an external source. The SegFlow autosampler consists of SegFlow 4800, SegMod, SampleMod 300, and associated accessories such as a FISP probe and cleaning liquid containers. The sample processing system consists of a µSIA, an SMA‐A micro‐volume flow cell, a DH‐2000 Ocean Insight deuterium lamp spectrophotometer, and a Protein A column.

### 2.2 PAT Instrument Modules

The integrated PAT system, composed of SegFlow, µSIA, and UPLC, is a fully automated platform configuration to achieve online sampling followed by titer measurement, in‐line sample preparation, and subsequent online UPLC analysis. The Flownamics SegFlow 4800 online sampling system is interfaced with a µSIA device to draw cell‐free sterile samples from the bioreactors using a 310 nm F‐series FISP probe with a 0.2 mm pore size ceramic membrane (Flownamics Inc., Madison, WI) to deliver samples to the µSIA through SegMod‐SampleMod. Such online sampling technology allows rapid and accurate sampling from up to eight bioreactors to deliver samples to a maximum of four analyzers. Thus, the existing offline and at‐line analyses are seamlessly integrated into an online PAT platform through FIAlab's SIAsoft software. The SIAsoft simultaneously acquires and exports all integrated data to any OPC‐enabled supervisory control and data acquisition (SCADA) for enhanced process monitoring and control. Custom scripts were written to establish communication between the SegFlow autosampler and the µSIA so that sample withdrawal and the subsequent workflow can be scheduled and coordinated with minimal human intervention.

Custom scripts were written in the Python programming language to streamline the end‐to‐end process with a fully automated configuration. The first step of the workflow begins with acquiring cell‐free culture samples from the bioreactor using SegFlow, followed by the loading of a fixed volume of the cell‐free sample onto a Protein A cartridge with UV detection at 280 nm for protein concentration determination. The second step repeats the first, but instead loads a fixed mass of protein onto a Protein A column at neutral pH and subsequent protein elution at a low pH. In the third step, the eluted protein is subjected to acid hydrolysis to release the sialic acid, followed by DMB labeling. DMB‐labeled sialic acid is then injected into the UPLC for the separation and quantitation of NANA and NGNA. The scheduling of sample withdrawal and sample analysis can be customized according to the user preference. The open‐source features enable feedback control via a distributed control system (DCS).

**FIGURE 1:**
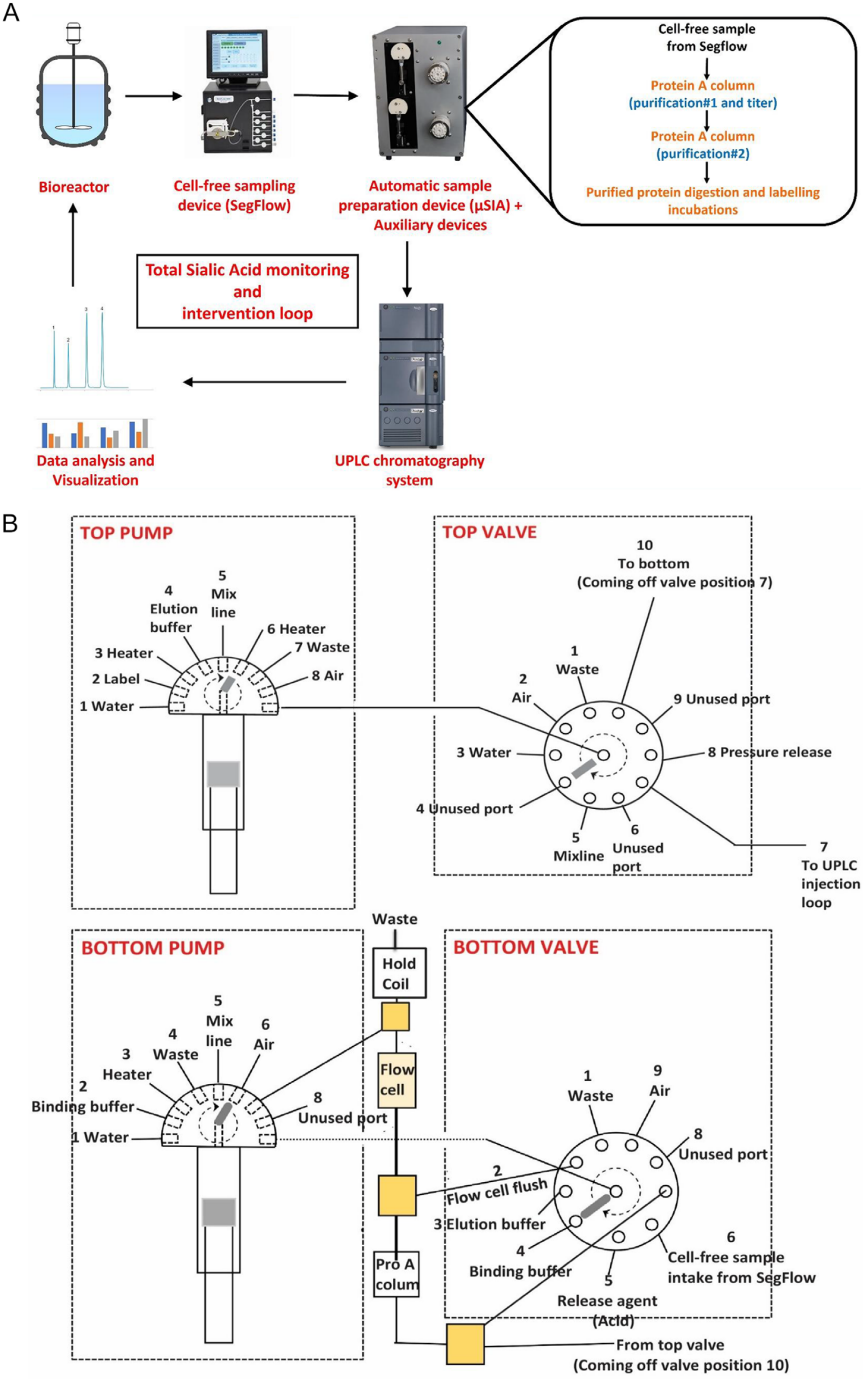
(A) Schematic of integrated µSIA system with Segflow and UPLC. (B) Schematic of µSIA system architecture.

The µSIA system interfaced with SegFlow and UPLC (Figure 1A) has served as the online PAT tool for measuring sialic acid concentrations of proteins from a 5L bioreactor (Applikon, Foster City, CA) to maintain the desired levels of sialic acid across all batches. The Lab‐On‐Valve µSIA system consists of a fully integrated 10‐position selector valve, enabling high reproducibility of the workflow, including sample preparation, mixing, injection, and optical monitoring in an automated fashion, with all reaction chemistry occurring within the valve manifold, eliminating the need for additional tubing and connectors. As depicted in Figure 1B, each of the Lab‐On‐Valve ports is assigned a specific function such as flow‐through, reagent aspiration, composite sample handling, and waste elimination. The individual ports are interconnected by microchannels and a built‐in multipurpose flow cell that is interfaced with optical fiber probes for spectral analysis. This fully automated system replaces otherwise laborious manual sample preparations.

The system uses a computer‐controlled multi‐position valve and a peristaltic pump and operates synchronously with this valve. In µSIA, samples and reagents are aspirated into the holding coil by operating the pump in reverse mode such that the carrier is returned to the reservoir. The restoration of forward pumping is synchronized with the opening of the valve port leading to the detector. The flow reversal leads to a mixing of the stack of sample and reagent zones to form a product zone, which is transported to the detector. The pump tubing comes into contact only with the carrier while the sample and reagents are being aspirated into the holding coil. The µSIA can be fully automated by coupling it with two 10‐port valve manifolds, two nine‐port syringe pumps, and computer control. This automation allows for the incorporation of wet lab procedures with precise control of assay parameters, including sample dilution, sample addition, mixing, incubating, and other specific parameters. The µSIA consists of a reaction coil, high‐precision bi‐directional syringe pumps (SP1 and SP2), and a peristaltic pump. The peristaltic pump, furnished with PTFE tubing, is utilized for filling the conduit from the external sample reservoir if SegFlow is not being used for sampling.

## 3 Experimental

### 3.1 Online Sampling, Inline Protein A Purification, Sialic Acid Release, DMB Derivatization

For online sampling from bioreactors, the µSIA system interfaced with the SegFlow autosampler draws samples from the bioreactor and subsequently delivers the sample to the designated port of the µSIA module. The received sample is then loaded onto a Protein A column and eluted in a fixed volume of elution buffer to obtain the protein titer of the bioreactor sample. The eluted protein that passes through the UV detector is sent to the waste line during the first cycle. Based on the titer information generated during the first cycle, a predetermined quantity of protein is loaded onto the Protein A column during the second cycle and subjected to inline Protein A purification to remove process impurities from the protein of interest. The purified protein is then subjected to acid hydrolysis to release the sialic acid and subsequently labeled with DMB. The DMB‐labeled sialic acid is then injected into the UPLC for analysis to separate NANA from NGNA and other components of the reaction mixture. Picomoles of NANA and NGNA are determined by interpolating the peak responses from the respective standard curves constructed using the DMB‐labeled NANA and NGNA standards. From the calculated picomoles of NANA and NGNA, the mole/mole ratio of sialic acid to protein is determined based on the titer value generated during the initial Protein A step described above.

### 3.2 Initial Evaluation of Chromatographic Conditions to Separate NANA and NGNA

Two Ascentis Express 90 Å RP‐amide columns (10 cm × 2.1 mm, 2.7 µm) were initially evaluated under isocratic conditions using a mobile phase of 0.1% formic acid in 10% acetonitrile for 10 min at a flow rate of 0.2 mL/min. Column temperature was maintained at 30°C. An experimental run was performed with an NANA and NGNA standard mixture at a 1:1 ratio.

### 3.3 Optimized Chromatographic Conditions to Separate NANA from NGNA

To separate labeled sialic acids (NANA and NGNA) from multiple components of the reaction mixture and resolve NANA from NGNA, columns of different chemistries, including the Agilent C18 column (Infinity Lab Poroshell 120 EC‐C18, 2.1 × 75 mm, 2.7 µm, narrow bore LC column), Supelco C‐18 column, Waters X‐Bridge column, and Sigma/Aldrich Ascentis Express RP‐Amide column (2.7 µm, 10 cm × 2.1 mm) were evaluated. Each column was evaluated using various gradient conditions and column temperatures.

### 3.4 Acid Hydrolysis versus Sialidase Digestion

To execute an automated workflow using µSIA, the Protein A purified sample from the collection coil is subjected to de‐sialylation and DMB labeling. To release sialic acid from glycoprotein, acid hydrolysis and sialidase digestion were evaluated. To release sialic acid using sialidase (Agilent kit), 18 µL of sample + 4 µL of Sialidase A + 8 µL of reaction buffer were incubated at 37°C for 30 min, followed by 3 h of labeling with DMB. For acid hydrolysis, 90 µL of the sample was treated with 10 µL of phosphoric acid and incubated at 80°C for 2 h, followed by 3 h of DMB labeling. After labeling, the volume was adjusted to 1 mL with water, and the entire mixture was collected in a collection vial.

The HPLC delivery line is flushed with 1000 µL of water and then primed with 800 µL of labeled sample. Then, 200 µL of labeled sample is delivered into the retention vial if needed to repeat the analysis. The syringe pump empties the remaining 600 µL of sample into the HPLC delivery line such that 300 µL primes the line and is sent to waste. The next 10 µL stays in the LC sample loop and is preserved for injection into the UPLC system under online analysis mode. The rest of the labeled sample is directed to waste after the 10 µL sample is injected onto the column, and the loop valve is switched back to the original position.

### 3.5 Attempt to Reduce Acid Hydrolysis Time Comparable to Sialidase Digestion of 30 min

A comparison of acid hydrolysis and sialidase digestion indicated that sialic acid release was higher with acid hydrolysis, despite the inefficiency of its longer digestion time. To reduce the 2‐h acid hydrolysis time to make it comparable with the 30‐min sialidase digestion time, an experiment was conducted to evaluate 30‐min acid hydrolysis versus 2‐h acid hydrolysis at 37°C. Analysis was conducted in offline mode.

### 3.6 Optimization of DMB Derivatization Time

To optimize DMB derivatization, a time‐course study was carried out at 50°C for 2, 3, and 4 h. For DMB labeling, 30 µL of sample and 10 µL of labeling reagent were vortexed and incubated at 50°C for 3 h. After incubation, 160 µL of water was added, and the mixture was transferred to HPLC vials for offline testing.

### 3.7 One Factor at a Time (OFAT) Analysis to Optimize Sialic Acid Release Derivatization

To optimize the desialylation and DMB derivatization, a screening of OFAT statistical analysis was conducted. OFAT was chosen over DOE (design of experiment) to identify the preferred course of action for sialic acid release (sialidase digestion or acid hydrolysis) and DMB derivatization. The OFAT analysis included a time course study to select the optimal combination of de‐sialylation and DMB derivatization conditions.

### 3.8 Fully Integrated SegFlow/µSIA/UPLC Platform

With the help of an appropriate information management system, synchronization between µSIA and UPLC can be fully established with or without the integration of a distributed control system. When the method run is initiated from µSIA, the software immediately sends a command to execute an inject hold state on the UPLC. Subsequently, when starting the established UPLC sequence, the LC injection valve is switched to the Load position so that the LC can accept the sample from µSIA. While the sequence is formally initiated, the Inject Hold status of the FIAlab method prevents the system from proceeding to the injection at this point. When the µSIA has processed the sample and filled the LC injection loop, the inject hold command will be released, and the LC will now proceed with the first injection in the sequence. The fully integrated architecture consists of SegFlow for online sampling, µSIA for automated sample preparation, and UPLC for sialic acid profiling. This integrated system facilitates the execution of a fully automated platform for timely measurements of sialic acid directly from upstream bioreactors and downstream AKTA purification systems.

## 4 Results

### 4.1 Inline Protein A Purification

During the Protein A purification step, an issue was encountered with the appearance of an interfering shoulder on the chromatogram, derived from the stroke of the syringe pump during the delivery of the elution buffer. This artifact was impacting the accurate titer determination. To overcome this issue, hardware and Python scripts were modified to deliver the elution buffer from two syringes: one from the top module and the other from the bottom module of the µSIA system. With this modification, the artifact issue was resolved, and a fully automated system configuration was enabled with the advantage of generating online titer values required for reporting of the accurate mole‐to‐mole ratio of sialic acid to protein.

### 4.2 Initial Evaluation of Chromatographic Conditions to Separate NANA and NGNA

**FIGURE 2:**
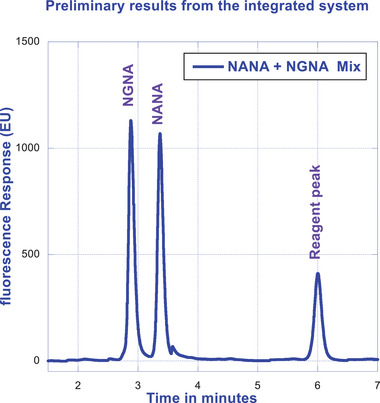
Chromatographic profile of NANA and NGNA under isocratic run.

Initially, we encountered an issue with the absence of synchronization between µSIA and the UPLC injector to trigger the injection upon receiving the inject command from the µSIA. Neither changing the Python script nor modifying the instrument settings had enabled the synchronization of the UPLC injection in response to receiving the command from the µSIA. To overcome this challenge, an external valve was mounted onto the UPLC system. The external valve acts as a switch between the μ SIA and the UPLC system to accept the command from the event table of the instrument method. With this hardware modification, the communication between µSIA and UPLC has been fully enabled. Figure 2 exemplifies the progress made on the synchronization, exhibiting a representative online chromatogram of NANA and NGNA, generated using Ascentis RP‐amide column with the use of mobile phase 0.1% FA/10% acetonitrile under an isocratic run at 30°C for 10 min at a flow rate of 0.2 mL/min.

### 4.3 Optimized Chromatographic Conditions to Further Separate NANA from NGNA

**FIGURE 3:**
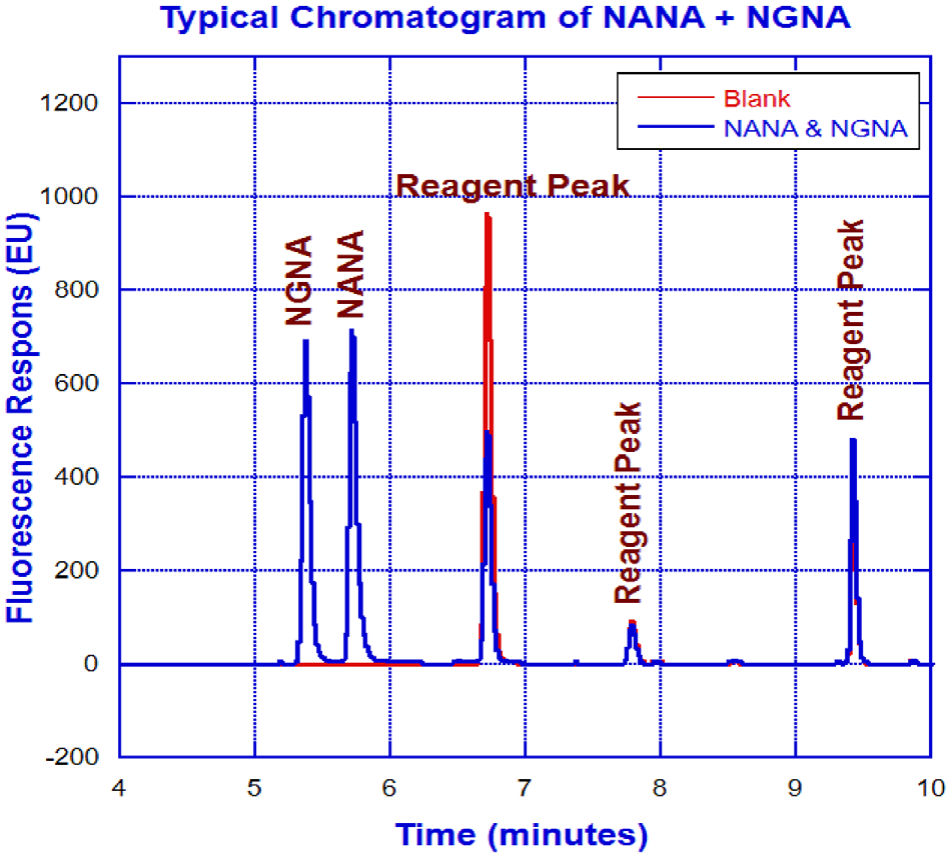
Chromatographic profile of NANA and NGNA under gradient run.

**FIGURE 4:**
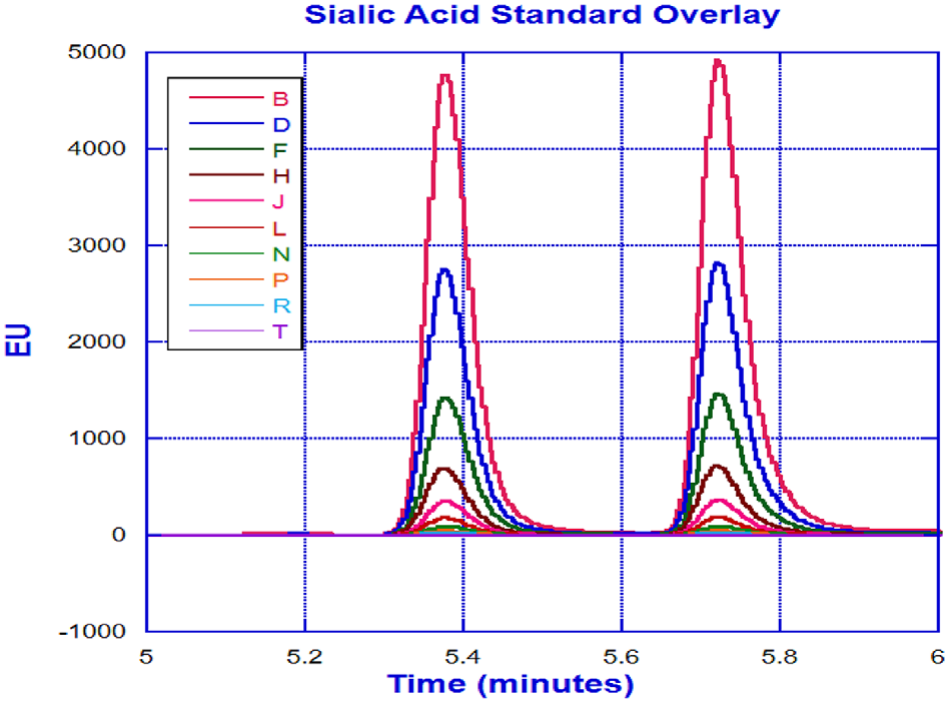
Overlay of NANA and NGNA standards at different concentrations (B, D, F, H, J, L, N, P, R, and T denote 1000, 500, 250, 125, 62.5, 31.25, 15.6, 7.85, 3.9, and 1.9 picomoles, respectively).

Despite the encouraging outcome from the initial evaluation of the Ascentis RP‐amide column, further optimization was needed to improve the resolution between NANA and NGNA, preferably under a gradient condition. We evaluated columns from other manufacturers against Ascentis RP‐amide column to make the best possible selection. As a mixed mode column, Ascentis RP‐amide column outperformed other columns, and hence the decision was to move forward with Ascentis RP‐amide column for further optimization. To achieve better separation between NANA and NGNA, a gradient elution instead of an isocratic run was carried out at 0.2 mL/min flow rate by maintaining the column temperature at 30°C. Mobile phases used for the gradient runs were 0.1% formic acid in water and 0.1% formic acid in acetonitrile as mobile phases A and B, respectively. An initial gradient of 6% was maintained for 1 min followed by a ramp up to 20% for 3 min and a subsequent 2‐min isocratic run. The eluate is detected with a fluorescence detector (excitation and emission wavelengths are 373 and 448 nm, respectively). As illustrated in Figure 3, the chromatogram obtained from the finalized condition has exhibited baseline‐separated NANA and NGNA. Overlay of NANA and NGNA standards at different concentrations is depicted in Figure 4.

### 4.4 Acid Hydrolysis

#### 4.4.1 Sialidase Digestion

**FIGURE 5:**
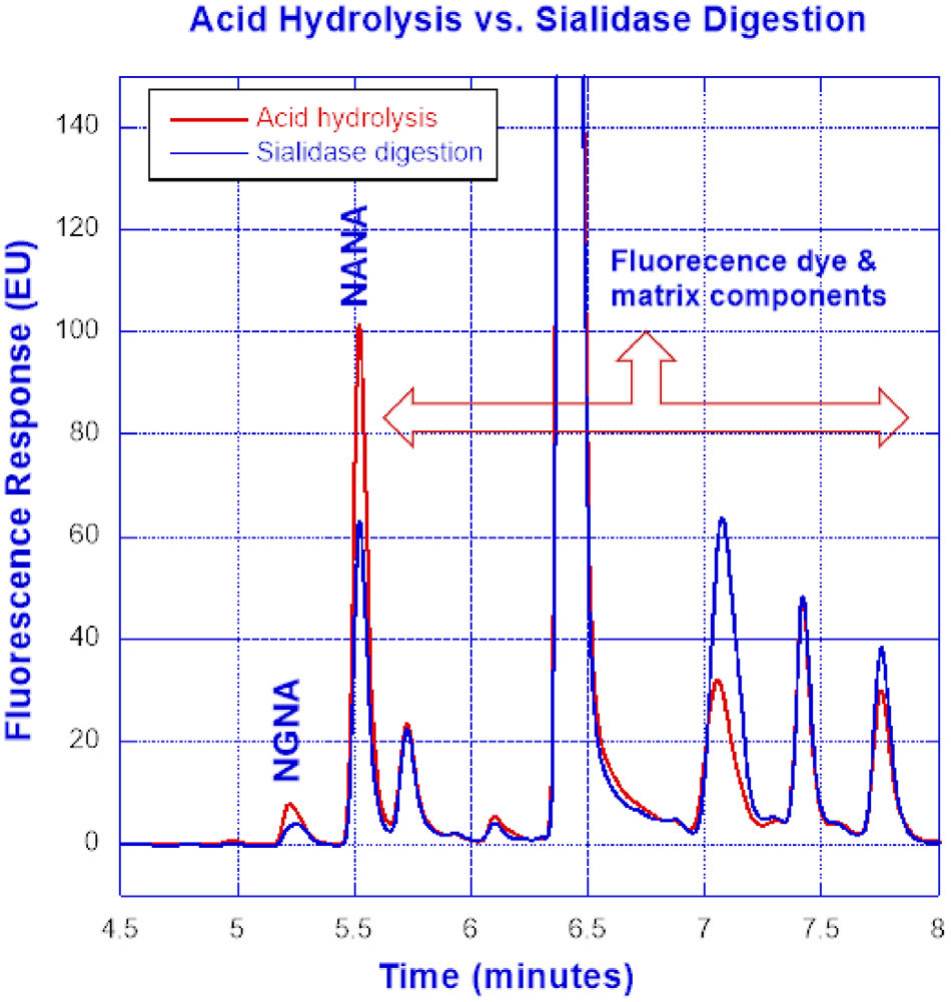
Overlaid chromatograms resulted from acid hydrolysis versus sialidase digestion.

To fully execute the automation of acid hydrolysis, a hardware reconfiguration was required. Although the PNGAse‐F digestion script established by FIA labs for N‐glycan release can be adapted for sialidase digestion to release sialic acid, the same script was not applicable for acid hydrolysis. While the PNGase‐F and sialidase digestions can be carried out within the column in conjunction with protein A purification, the protein A purification and acid hydrolysis needed to be decoupled for acid hydrolysis as the low pH environment of acid hydrolysis is incompatible with the binding of the protein to the Protein A column. While PNGase‐F and sialidase digestion is taking place in the presence of bound protein on the column at 37°C, the same approach cannot be adapted for acid hydrolysis due to the negative impact on binding and column integrity under the highly acidic condition. The decoupling of protein A purification and hydrolysis had presented another challenge of not having a heater to perform the hydrolysis at 80°C for 2 h. To overcome this challenge, a hardware reconfiguration was enabled such that the column heater from the Protein A compartment was relocated to perform hydrolysis at a higher temperature as it is acceptable to do Protein A purification at ambient temperature. Evaluation results of acid hydrolysis versus sialidase digestion suggested that acid hydrolysis is more efficient than sialidase digestion. As shown in Figure 5, the blue line is from the 30‐min sialidase digestion and 3 h labeling of Protein‐X, and the red line is 2 h acid hydrolysis and 3 h labeling of the same protein.

### 4.5 Attempt to Reduce Acid Hydrolysis to 30 min (Same as Sialidase Digestion)

Although acid hydrolysis outperformed sialidase digestion, the time for acid hydrolysis is significantly longer (2 h), compared to 30 min for sialidase digestion. An attempt made to reduce the hydrolysis time to 30 min to make it comparable to sialidase digestion was not successful. It was demonstrated that 30‐min hydrolysis is not as effective as 2‐h hydrolysis. The intensities of NANA and NGNA peaks were significantly lower for 30‐min hydrolysis (data are not shown).

### 4.6 Optimization of DMB Derivatization Time

**FIGURE 6:**
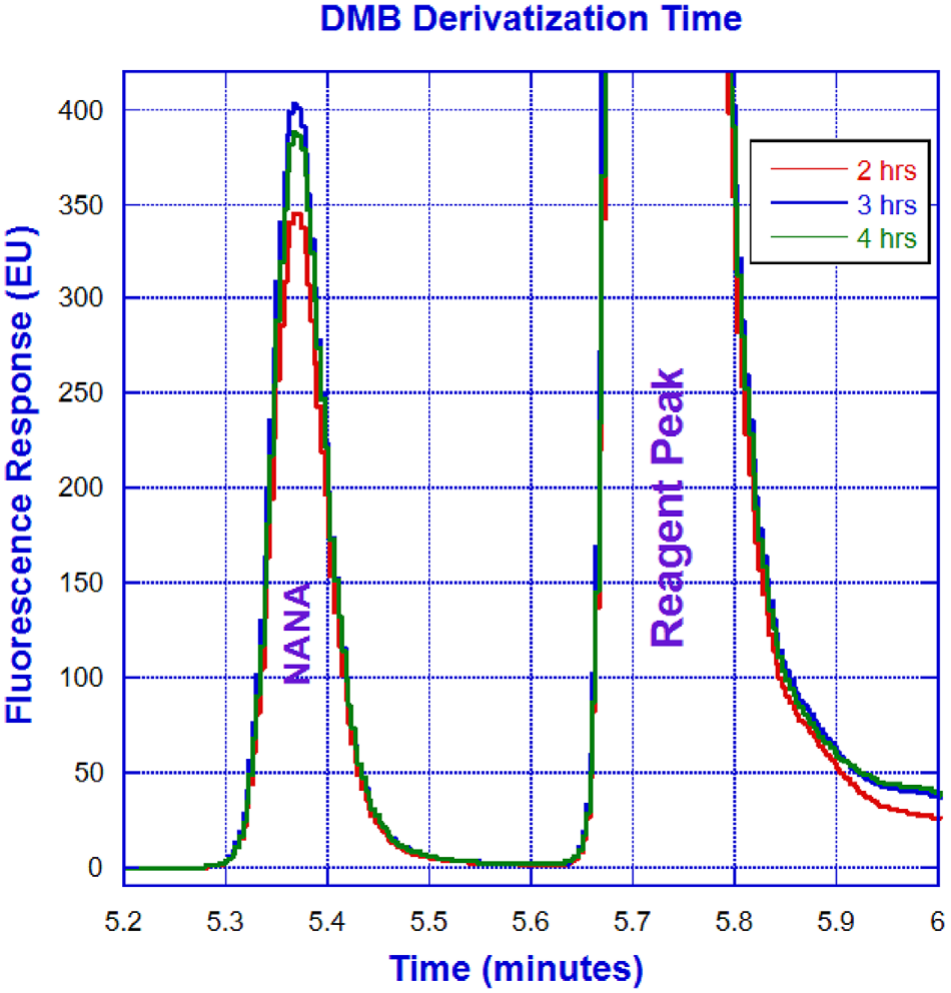
Comparison of 2, 3, and 4 h of DMB derivatization time.

A time course study conducted to optimize DMB derivatization has suggested that maximum derivatization is achieved at 3 h, which is in perfect agreement with the recommendation furnished by Agilent, the sialic acid kit provider. As depicted in Figure 6, the peak intensity is relatively higher for 3‐h labeling in comparison to 2‐ and 4‐h counterparts. The thermal degradation of NANA (decarboxylation and deacetylation) that occurs with prolonged heating at low pH may explain the lower recovery for 4‐h incubation. A combination of lower pH and higher temperature along with longer incubation time can increase the degree of degradation [32]. In addition, the DMB labeling process often involves extremely low pH, which can destabilize the labeled sialic acid and cause loss of fluorescence intensity during longer incubation.

### 4.7 OFAT Analysis to Optimize Sialic Acid Release and Derivatization

OFAT experimental results generated with sialidase digestion, acid hydrolysis, and DMB labeling at different time courses have suggested that 2‐h acid hydrolysis in conjunction with 3‐h DMB labeling is the optimal combination for the best performance. Also indicated that the RP‐Amide column provided better recovery of NANA. OFAT results are presented in Table 1.

**TABLE 1:** Results of one factor at a time (OFAT) analysis.

Digestion conditions (*n* = 4)	RP amide column		C18 column	
	NGNA (mol/mol)	NANA (mol/mol)	NGNA (mol/mol)	NANA (mol/mol)
2‐h sialidase digestion 3‐h labeling	0.45 ± 0.03	6.00 ± 1.10	0.42 ± 0.01	5.80 ± 0.64
1‐h acid digestion 3‐h labeling	0.50 ± 0.02	6.45 ± 0.61	0.47 ± 0.05	6.25 ± 0.24
2‐h acid digestion 2‐h labeling	0.50 ± 0.02	6.68 ± 0.94	0.50 ± 0.02	6.42 ± 0.54
2‐h acid digestion 3‐h labeling	0.62 ± 0.02	8.06 ± 0.79	0.57 ± 0.06	7.58 ± 0.30
2‐h acid digestion 4‐h labeling	0.63 ± 0.03	7.94 ± 0.46	0.59 ± 0.07	7.49 ± 0.09

### 4.8 Fully Integrated SegFlow/µSIA/UPLC Platform

**FIGURE 7:**
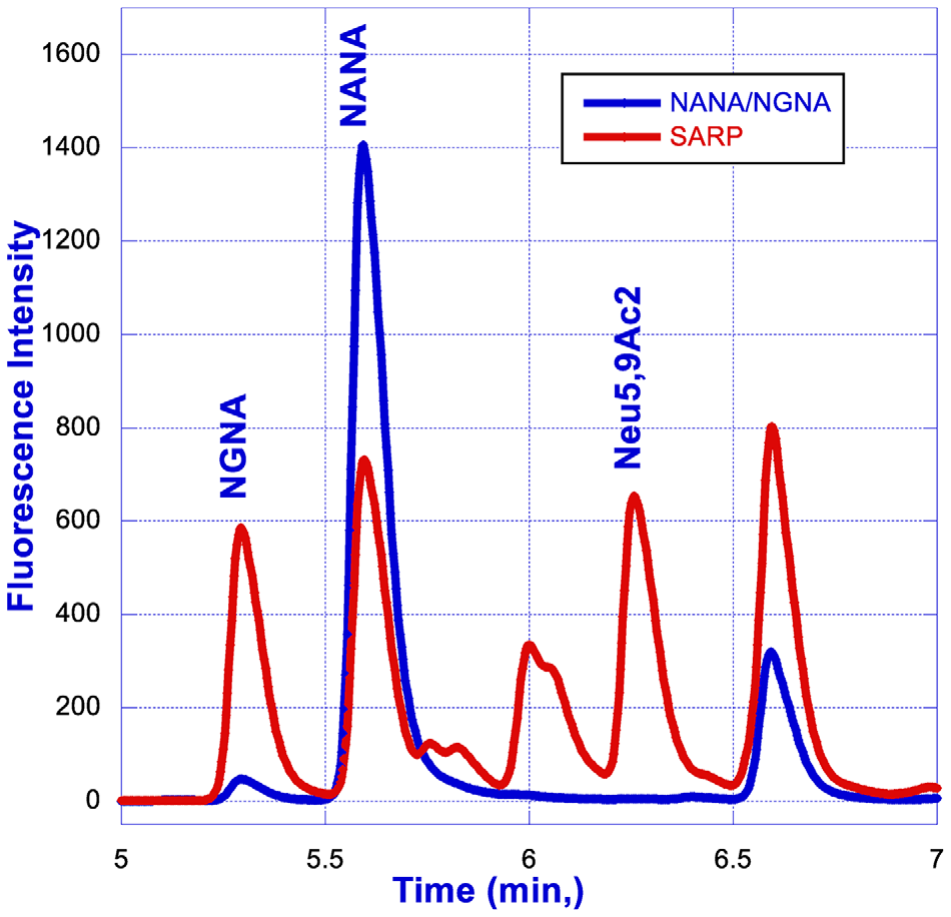
Overlay of NANA/NGNA and sialic acid reference panel (SARP).

**FIGURE 8:**
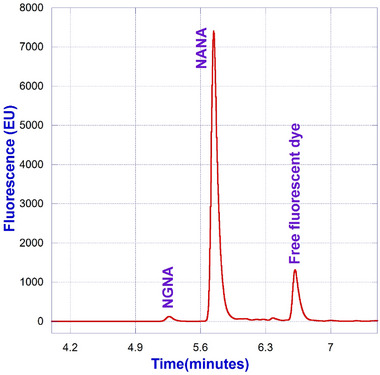
A representative chromatogram of Protein‐X reference material.

A fully integrated workflow consists of the following steps. Samples diluted 1:1 with water are subjected to an acid hydrolysis at 80°C for 2 h to release the sialic acid from the intact protein. Subsequently, the released sialic acid is labeled with DMB at 50°C for 3 h. The DMB‐labeled sialic acid is then quenched with 60 µL of water prior to the subsequent step of UPLC analysis. For the UPLC analysis, an RP‐amide column is leveraged with the utilization of 0.1% formic acid in water and 0.1% formic acid in acetonitrile as mobile Phases A and B, respectively. Fluorescence detection of the eluate was carried out at an excitation wavelength of 373 nm and an emission wavelength of 448 nm. Figure 7 represents a typical chromatogram of a sialic acid standard mixture containing NANA, NGNA, and Neu5, 9Ac2. A representative chromatogram of DMB‐labeled sialic acid released from Protein‐X using the optimized workflow is shown in Figure 8.

**FIGURE 9:**
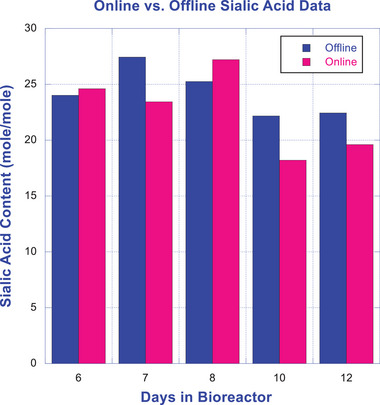
Comparability of offline versus online sialic acid data illustrated in bar graph.

As shown in Table 2 and Figure 9, the offline data generated using the conventional approach and the online data generated using the integrated SegFlow/µSIA/UPLC platform are quite comparable with minor differences that are within the inherent variability of the UPLC‐based sialic acid method. Please note that the comparability study of the online versus offline method was executed at *n* = 1 due to the limitation of having only a single online system in our lab. Sequential analyses of online samples present the challenge of inconsistency and inaccuracy due to the dynamically changing environment of the cell culture sample in the bioreactor. To demonstrate the precision of the online method, a pseudo bioreactor run of a cell‐free Day‐7 bioreactor sample was carried out. The Day‐7 bioreactor sample was manually collected and subjected to cell removal to arrest cell growth and apoptosis before undergoing online analysis in 5 replicates. The data presented in Table 3 demonstrate that the %RSDs are 1.8 and 5.3, respectively, for the offline and online methods, which is within expectation, considering that offline data was intra‐assay based (batch testing) while online results were generated with individual runs under the inter‐assay mode.

**TABLE 2:** Comparability of offline versus online sialic acid data of bioreactor samples.

No. of days in bioreactor	Offline data			Online data		
	NGNA	NANA	TSA	NGNA	NANA	TSA
6	0.02	24.01	24.03	0.02	24.59	24.61
7	0.03	27.43	27.45	0.02	23.45	23.45
8	0.02	25.24	25.26	0.03	27.18	27.21
10	0.02	22.15	22.17	0.02	18.20	18.21
12	0.02	22.40	22.43	0.02	19.59	19.61

**TABLE 3:** Precision study (*n* = 5) using Day 7 sample of bioreactor run.

*N*	Offline data			Online data		
	NGNA	NANA	TSA	NGNA	NANA	TSA
	(mol/mol)	(mol/mol)	(mol/mol)	(mol/mol)	(mol/mol)	(mol/mol)
1	0.03	24.15	24.18	0.03	25.11	25.14
2	0.03	23.25	23.27	0.03	27.70	27.73
3	0.03	24.06	24.09	0.03	27.15	27.18
4	0.03	23.68	23.71	0.02	23.94	23.96
5	0.03	23.06	23.08	0.03	26.32	26.35
Average (*n* = 5)	0.03	23.64	23.67	0.03	26.04	26.07
SD	< 0.01	0.43	0.43	< 0.01	1.37	1.37
%RSD	NA	18	1.8	NA	5.3	5.3

### 4.9 Summary of Data Turnaround Time (Online versuss Offline)

As depicted in Table 4, the data turnaround time for the PAT‐based offline method is around 24 h, while it is about 3‐fold lower for the online method. The offline workflow is complex, consisting of sampling, aliquoting, forecasting, material transfer, purification, UPLC‐based titer measurement, acid hydrolysis, DMB derivatization and chromatographic separation, data processing, data review and approval, and data communication, which can take anywhere between 24 and 48 h. On the other hand, the data turnaround time for online method can be greatly reduced with the utilization of a fully integrated SegFlow‐µSIA‐UPLC platform with the pursuit of online sampling, inline purification, automatic sample preparation, and online UPLC analysis, as well as automatic data processing, digitalization and data synchronization by harnessing software packages such as L7 informatics’ Unified Enterprise Science Platform (ESP).

**TABLE 4:** Summary of turnaround times for online and offline sialic acid methods.

Analysis steps	Turnaround time (Offline)	Turnaround time (Online)
Sample collection, forecasting, scheduling, sample delivery and receiving	8 h	10 min
Protein purification	2 h	10 min
Titer measurement	4 h	10 min
Acid hydrolysis	2 h	2 h
DMB derivatization	3 h	3 h
UPLC analysis	20 min	20 min
Data processing and reporting	5 h	10 min
Turnaround time	∼24 h	∼6 h

## 5 Discussion

Sialic acid capping at the nonreducing terminals of N‐ and O‐glycans can have an impact on biological actions in mediating efficacy, immunogenicity, PK, and PD of therapeutic glycoproteins. Depending upon the nature of the molecule, the terminal sialic acid may play diverse roles in impacting the rate of clearance, inflammatory response, and antibody‐dependent cellular cytotoxicity (ADCC). Of the NANA and NGNA isotypes found in biotherapeutics, NGNA can potentially be immunogenic to humans, as this isoform is not synthesized by humans. For this reason, monitoring and controlling the levels of NGNA present in therapeutic glycoproteins from a safety perspective is as critical as monitoring and controlling NANA for achieving desired PK. To maintain the desired levels of NANA and NGNA, the time of upstream harvest decision is often determined based on the levels of sialic acid derivatives present. Similarly, there is an opportunity to control sialic acid content during the downstream polishing step by adjusting the ionic strength of the wash buffer.

To monitor sialic acid levels within the specified range, the current practice of submitting samples for offline analysis with a prolonged waiting period is inefficient and raises concerns about the validity of the time‐point. This lengthy lag‐time risks missing the optimal harvest time‐point at which sialic acid peaks. Once peak levels are reached, a rapid decline in sialic acid content is expected due to increased sialidase activity associated with apoptosis. Therefore, real‐time or near real‐time sialic acid measurements are essential. Previous attempts (data not shown) to utilize spectroscopic techniques for predictive modeling and real‐time data acquisition had limited success. To maintain sialic acid measurements comparable to the currently deployed offline UPLC method, we automated the workflow, including bioreactor sample withdrawal, protein A purification, acid hydrolysis, DMB labeling, and automated delivery of labeled sialic acid to the UPLC.

To establish an automated integrated workflow, the SegFlow autosampler was interfaced with a µSIA system at the front end and a UPLC system at the back end. The samples drawn from the bioreactors are deposited in a sampling tube located in the µSIA module. Subsequent injection of the sample from the tube to the protein A cartridge allows online auto‐purification in conjunction with automatic titer measurement. Protein A purified samples are then subjected to acid hydrolysis and DMB derivatization prior to injection into the UPLC system, equipped with an RP‐amide column and fluorescence detector. NANA and NGNA, which are separated from other components as well as from each other, are then quantitated against their respective standard curves. The quantitation values of mole/mg are then converted to mole/mole ratios.

As illustrated in Figure 1, the integrated µSIA platform is capable of automatic sample preparation with the help of tailored Python scripting. Operating parameters and scripts were optimized for each module of the system for the best overall performance. For protein A purification, 0.02 mL columns from FIA labs, 1.0 and 0.5 mL columns from Cytiva, and 0.5 and 0.25 mL columns from RepliGen were evaluated. From a practical perspective, a 0.25 mL column from RepliGen was chosen as it can provide the right amount of purified material within the scope of the existing instrument architecture and infrastructure. For the UPLC separation of NANA and NGNA, the initial evaluation carried out under an isocratic run provided separation of NANA and NGNA (Figure 2) but required further optimization. As shown in Figures 3 and 4, baseline separation of DMB‐labeled NANA and NGNA was achieved under the optimized gradient condition. As shown in Figure 5, acid hydrolysis outperformed sialidase digestion. An attempt to shorten the hydrolysis time from 2 h to 30 min was not successful (figure not shown). As depicted in Figure 6, the chromatographic profiles of DMB derivatization carried out at different time courses suggested that optimal labeling is achieved at 3 h. Table 1 showcases the results of DOE screening (one‐factor‐at‐a‐time analysis) of two different columns, as well as a time‐course study of acid hydrolysis and DMB labeling. The results suggest that the RP‐amide column outperformed the C18 Poroshell column. Optimal acid hydrolysis and DMB derivatization times were determined to be 2 h and 3 h, respectively.

This novel approach presented several roadblocks that required resolution. To establish precise online sialic acid results comparable to the legacy offline results, we modified the Python scripting and hardware configurations. These modifications ensured proper sample delivery between modules with 100% transferability, as even minor sample loss or inline dilution could negatively impact results. Therefore, we took a highly orchestrated approach to hardware reconfiguration and Python scripting to eliminate these potential issues. To overcome inline dilution, we incorporated purging of each line with its respective sample into the script. Additionally, we generated higher sample volumes, aspirated larger liquid volumes during transfers, and purged lines with air to displace residual liquids.

The integrated portable µSIA unit can be moved around to draw samples from the bioreactors, generating near real‐time sialic acid quantitation results. Online UV measurement of a protein A purified Fc‐containing protein is a critical step in determining the exact concentration of the analyzed samples. This accurate concentration is crucial for reporting the mole‐to‐mole ratio of sialic acid to protein. Initially, accurate UV measurement was difficult due to the default settings. An interfering shoulder appeared on the chromatogram, resulting from the syringe pump's upstroke and downstroke during the four rounds of elution buffer delivery. To overcome this challenge, hardware modification and the utilization of a smaller column were necessary. The column size was reduced from 1.0 to 0.25 mL. Additionally, the elution buffer was delivered from two syringes through a T‐connector, one from the µSIA's top module and the other from the bottom module. These modifications enabled a fully automated system configuration. This automation provides the advantage of generating online protein determination, which is required for reporting the mole‐to‐mole ratio of sialic acid to protein. As shown in Table 2 and Figure 9, online and offline results generated with bioreactor cell culture samples from Day 6 through Day 12 are comparable, with minor differences that fall within the inherent variability of the UPLC‐based sialic acid method.

## 6 Conclusion

In conclusion, sialic acid moieties of certain glycoprotein therapeutics influence the biological and physicochemical properties, thus impacting clinical performance. For certain molecules, slight variations in sialic acid content can significantly impact PK/PD. Hence, sialic acid is designated as a CQA for such biotherapeutics and is an essential regulatory requirement to monitor and control it within a specified range. To maintain consistent levels of sialic acid with reduced variability, harvest decisions are often based on sialic acid content. The current paradigm of offline testing with prolonged turnaround times conflicts with the need for rapid sialic acid results for timely harvest decisions, as well as for determining the conductivity of the wash buffer used during the downstream polishing step to control sialic acid levels. The online method described in this manuscript is intended to acquire near real‐time sialic acid data to facilitate timely process decisions. With the integrated approach described in this manuscript, both upstream harvest decisions and downstream wash buffer strength can be determined based on the near real‐time measurements of sialic acid using an integrated Segflow‐µSIA‐UPLC platform.

## Author Contributions

The authors listed in the manuscript have contributed their fair share based on their respective roles and responsibilities in the organization. Letha Chemmalil conceived and developed the vision and strategy for the project and wrote the paper. Tanmay Kulkarni developed hardware optimization and Python scripting for the microfluidic sialic acid analyzer (µSIA). He also took a lead in collecting and analyzing data. Mathura Raman developed and optimized the ultra performance liquid chromatography (UPLC)‐based sialic acid method and established its integration with the µSIA. Priya Singh developed a seamless integration between the µSIA, SegFlow, and UPLC. Chris Chumsae, Julia Ding, Gloria Li, and Anthony Leon provided analytical leadership at various stages of the project. Yueming Qian was an initial collaborator who provided strategic guidance and representative samples for establishing proof of concept. Kyle McHugh and Zhuangrong Huang from upstream process development were engaged in the actual execution of the integrated process analytical technology (PAT). Their work interfaced with upstream bioreactors to perform online testing during the bioreactor run. McHugh and Huang received guidance and leadership from Eric Hodgman and Michael Borys.

## Data Availability

All data presented in this manuscript and the source data are securely stored in Electronic Lab Notebook (ELN) and Empower Chromatography Data System (CDS), respectively. All data can be reliably retrieved in the future.
